# Five Strategies for a Safer EHR Modernization Journey

**DOI:** 10.1007/s11606-023-08331-z

**Published:** 2023-10-05

**Authors:** Dean F. Sittig, Edward E. Yackel, Hardeep Singh

**Affiliations:** 1 Department of Clinical and Health Informatics, McWilliams School of Biomedical Informatics, University of Texas Health Science Center at Houston, Houston, TX USA; 2 U.S. Department of Veterans Affairs, National Center for Patient Safety, Ann Arbor, MI USA; 3 Center for Innovations in Quality, Effectiveness and Safety, Michael E. DeBakey Veterans Affairs Medical Center, Houston, TX USA; 4 Department of Medicine, Baylor College of Medicine, Houston, TX USA

## Introduction

Safely transitioning to any new electronic health record (EHR) is difficult. Large-scale EHR transitions require more time, money, and effort and pose even greater risks to patient safety. EHRs control, constrain, or at a minimum interact with nearly every aspect of our complex adaptive healthcare system. In addition to surprises, problems, and delays,^1^ organizations transitioning EHRs often experience decreases in certain cost, quality, and efficiency measures for up to 2 years.^2^

While EHR software does not “wear out” like humans or machines, without a strong vision, robust underlying software architecture, singular governance structure, and constant innovation, it can become outdated and require replacement. The Department of Veterans Affairs’ (VA’s) CPRS/Vista product is nearing the end of its useful life. Absent a significant, multi-year, software re-write, it is unlikely to support the VA’s modernization efforts.

## Strategies For Safer EHR Transitions

EHR transition is a journey, not a destination. While the VA’s EHR transition is slated for 10 years, the work will not be over after the last site switches to the new EHR. EHRs are complex, software-controlled, electromechanical devices built and used by humans in unexpected ways. Both humans and these devices have known and unknown limitations and deficiencies. EHR transition, on-going usage, and never-ending maintenance require consistent, stable, committed leadership and constant vigilance. As with any long journey, problems and unexpected twists and turns must be managed. The journey’s success depends on the organizations’ ability to prepare for the worst, remain agile, identify obstacles, and then create, communicate, and implement solutions in a timely manner. Based on current literature and experiences, we recommend five actionable strategies to support the VA in a safer journey to EHR modernization. Operationalizing these strategies reduces the risk of EHR transitions inadvertently harming patients, causing substantial cost overruns, or leading to workforce turnover.

### Implement Shared Responsibility Principles for Safer EHR Transitions

Safer EHR transitions require shared responsibility between stakeholders including “vendors, care providers, provider organizations and their health IT departments, and public and private agencies focused on quality of care.”^3^ Organizations that pioneered EHR development will experience a significant change in the relationship with their EHR developer when they transition to a commercial, off-the-shelf EHR.^4^ Integrating shared responsibility principles into these relationships and day-to-day operations is essential for achieving safety and high reliability.

For example, addressing poor EHR usability requires significant, shared work by all involved—EHR vendors, health systems’ clinical and IT leadership, clinical and administrative users, and researchers. EHR vendors should be responsible for providing health systems with best practices they have identified across their customer base regarding EHR configuration, customization, and monitoring of end-user activity and system-to-system interface logs to identify problems. Over the longer term, EHR vendors need to work to improve their products by incorporating user-centered design processes and developing strategies for objectively measuring real-world usability. Simultaneously, health systems, such as the VA, need to ensure that their clinical content (e.g., orderable items, diagnostic codes, referral locations, clinical decision support, and charting templates) is complete and accurate and that users are properly trained. Clinicians and other staff need to participate in all training activities, use the EHR to perform their jobs completely and correctly, and report safety problems. Finally, informatics’ researchers should develop generalizable methods to monitor system functionality and conduct studies to identify best practices and mitigate usability-related safety hazards. Effective use of shared responsibility principles will not be easy but should lead to a positive safety culture, and a successful EHR transition.

### Reinvigorate, Empower, and Support Staff

A robust clinical and administrative staff is essential to maintain patient safety. Change becomes increasingly difficult for those feeling powerless to control their work environment. It is essential to have staff and leadership willingness and ability to invest time and effort required to make transitioning a success. Multi-directional communication with feedback loops is essential for success. For example, staff needs to hear why change is necessary, what changes are coming, when these changes will occur, and what they need to do to prepare for them. Staff also needs to hear about benefits the new EHR brings to the organization and how patient care and outcomes will improve. Conversely, leadership needs to hear from those being affected by the changes, including how changes are planned and implemented, what training is needed, what support is required, and what risks to safety may have emerged. Additional help and support should be instituted during the initial months following go-live. Short-term and intermediate “wins” can be recognized to boost morale.

### Standardize EHR Functionality and Workflows Whenever Possible

As Deming said, “Uncontrolled variation is the enemy of quality.” Several large, integrated healthcare delivery networks recently acknowledged that they needed to reduce unwarranted variation (i.e., variations not due to illness, medical need, or evidence-based medicine) in administrative and clinical care processes across their facilities to improve care quality and reduce costs.^5^ Identifying unwarranted variation requires active data collection, monitoring, analysis, and iterative process refinement to identify and implement best practices. Care must be taken not to standardize unsafe or inefficient processes. A key benefit of changing EHRs is the opportunity to re-think, reorganize, standardize, and streamline work across the VA. For example, standardizing how data are defined, collected, coded, stored, and corrected across the VA can ensure data interoperability within the VA and the Department of Defense and create a seamless EHR. This would facilitate care transitions, improve care quality, and increase the amount and quality of data available for analysis.

Developing a standard EHR build can reduce cost, time, and effort required to bring new VA sites live by reducing configuration, training, and maintenance activities. Moving from a distributed VA facility-centric care model where each facility does what is best for them to an enterprise care model that shares data, information, and knowledge across the VA will have substantial benefits in terms of cost, quality, service, employee well-being, equity, and safety.

### Optimize EHR Reliability, Response Time, and Design

To improve patient safety, VA’s EHR needs to be designed for safety, be easy to use, respond rapidly to user input, and be available 24 hrs/day, 7 days/week. System availability concerns must be addressed by eliminating single points of failure.^6^ Once the computing infrastructure is fast and reliably working, bolstering patient safety can be achieved using human-centered design principles to ensure that the EHR software is designed as intended, written as designed, working as expected, and being used completely and correctly by all users. Achieving safety thus requires extensive, end-to-end testing, first with a large number of users in a test environment configured as similarly as possible to the live environment. Testing should continue here until all serious safety issues are fixed. Additional testing with a small number of users in the live environment should then be undertaken to ensure no serious safety issues remain.^7^

EHR features, functions, user inputs, system-to-system interfaces, and error queues must be monitored closely. Monitoring can leverage manual or automated methods, such as manual retrospective EHR data queries to detect potential software glitches and automated real-time anomaly detection. Identified problems must be investigated and fixed expeditiously. Solutions should be crafted using a long-term perspective vs. a short-term workaround or “band-aid” mindset. Solutions should be easy to implement, learn, and use and rolled out and communicated as rapidly as possible to minimize risk and harm.

### Centralize EHR Safety Assessment, Measurement, and Monitoring

Ensuring safety of an enterprise EHR supporting a nationwide, integrated healthcare delivery system with over 1200 sites of care and 9.2 million patients is a daunting task. Such a challenge requires a centralized, dedicated entity with shared governance that is empowered to develop, implement, monitor, and evaluate safety-related surveillance and solutions. The entity should have multidisciplinary representation that includes expertise from clinical informatics, information technology, human factors, safety and quality science underpinned by measurement, cognitive science, social sciences, and multiple clinical disciplines. Distributing the responsibility to fix safety problems across different parts of the organization makes safety surveillance mechanisms less successful. This single entity must be provided with the people, time, and financial resources to do its job.

Continuous safety assessment, measurement, and monitoring should be a core function of this entity. This requires both proactive approaches, such as EHR safety risk assessment tools,^8^ and retrospective approaches, such as automated surveillance using a comprehensive set of EHR safety measures.^9^ The entity should also have authority to investigate and report on serious EHR safety events and proactively conduct random on-site EHR safety inspections.^10^ Additionally, this entity should work closely with the vendor to ensure their internal design, development, testing, and implementation processes lead to safe and effective products. Both the delivery system and EHR are continually changing; therefore, patient safety is dependent on a never-ending process of proactive safety and risk assessment, coupled with on-going error identification, categorization, investigation, prioritization, and remediation.

## Summary

We recommend five actionable strategies to support the VA in a safer journey to modernize the EHR. While these strategies are not exhaustive, they will reduce the risk of patient harm, cost overruns, and workforce turnover.
